# Energy Parameters and Novel Algorithms for an Extended Nearest Neighbor Energy Model of RNA

**DOI:** 10.1371/journal.pone.0085412

**Published:** 2014-02-21

**Authors:** Ivan Dotu, Vinodh Mechery, Peter Clote

**Affiliations:** 1 Biology Department, Boston College, Chestnut Hill, Massachusetts, United States of America; 2 Hofstra North Shore-LIJ School of Medicine, Hempstead, New York, United States of America; Ben-Gurion University, Israel

## Abstract

We describe the first algorithm and software, RNAenn, to compute the partition function and minimum free energy secondary structure for RNA with respect to an *extended nearest neighbor* energy model. Our next-nearest-neighbor triplet energy model appears to lead to somewhat more *cooperative* folding than does the nearest neighbor energy model, as judged by melting curves computed with RNAenn and with two popular software implementations for the nearest-neighbor energy model. A web server is available at http://bioinformatics.bc.edu/clotelab/RNAenn/.

## Introduction

Thermodynamics-based *ab initio* RNA secondary structure algorithms are used to detect microRNAs [1], targets of microRNAs [2], non-coding RNA genes [3], temperature-dependent riboregulators [4], selenoproteins [5], ribosomal frameshift locations [6], RNA-protein binding sites [7], etc. The importance and ubiquity of RNA thermodynamics-based algorithms cannot be overemphasized – there are even applications in RNA design for novel cancer therapies and in synthetic biology. Indeed, in [8] Vashishta et al. used the RNA minimum free energy (MFE) structure prediction algorithm mfold [9] to design seven anti-pCD ribozymes, four of which were cloned, stably transfected in the highly metastatic human breast cancer cell line, MDA-MB-231, and shown to have a therapeutic potential by knocking down the expression of pCD. (Procathepsin D (pCD) is correlated with highly invasive malignancies, such as breast cancer. Ribozymes, first discovered by the Nobel laureats, T. Cech and S. Altman, are RNA enzymes that can cleave a molecule or catalyze a reaction.)

Following pioneering work of the Tinoco Lab and Freier et al. [10], a number of increasingly sophisticated *nearest neighbor* models have been defined: INN [11], [12], INN-HB, also called *Turner99* [13], *Turner2004* [14], [15], as well as models that incorporate knowledge-based parameters [16], [17]. These free energy parameters of the *nearest neighbor* (NN) model form the foundation for essentially all current thermodynamics-based RNA algorithms: minimum free energy (MFE) secondary structure [9], [18], Boltzmann partition function [19], maximum expected accuracy secondary structure [20], MFE secondary structure with pseudoknots [21], sampling suboptimal structures [22], RNA sequence-structure alignments [23], etc.

Benchmarking studies have shown that, on average, the minimum free energy structure includes 73% of base pairs in X-ray structures when domains of fewer than 700 nucleotides (nt) are folded [24]; i.e. prediction *sensitivity* of the MFE structure is 73%, although accuracy drops as sequence length increases. There is increasing evidence that by improving the free energy parameters, structure prediction accuracy can be improved. Andronescu et al. [16] used combinatorial optimization to determine optimal weights 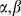 for which energy parameters are determined by 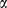-weighted contribution from Turner's free energies together with 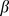-weighted contribution from knowledge-based potentials, the latter obtained from the negative logarithm of frequencies in existent structure databases. Free energy parameters in the Turner model are determined by a least-squares fit of UV absorption data based on the assumption that change in heat capacity, 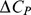, is zero. This assumption is erroneous, as pointed out by Mikulecky and Feig [25], who observed that the hammerhead ribozyme does not fold in 2-state transition, but rather has 3 states: cold denatured, folded and hot denatured. In [17] M. Bon improved MFE structure prediction by defining new parameters for the nearest neighbor model that account for linear dependence of change 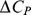 of heat capacity on sequence length and by incorporating knowledge-based potentials from a hand-curated selection of Sprinzl's transfer RNA database [26].

### Subsection 1.1: Motivation from protein helix-coil transition

Consider a coarse-grain classification of amino acids, where a polypeptide chain is given by an 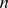-mer, or length 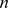 sequence 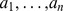 of amino acids, where each residue 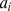 is either in an H (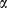-helix) or C (coil) conformation. Assume that the energy of an 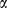-helical residue is 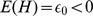, while that of a coil residue is 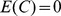. A protein with many residues in an 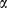-helical conformation at room temperature, such as hemoglobin, will unfold into a random coil at a higher temperature, where all previous H residues have been transformed into C residues. In particular, if 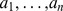 is an 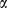-helix, then at low temperature, all residues are H, while at high temperature all residues are C. The partition function 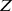 of 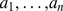 is defined by 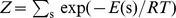, where the sum is taken over all 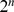 many sequences 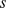 of H's and C's. Using the (temperature-dependent) partition function, we can compute the expected number 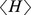 of 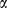-helical residues for the 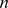-mer 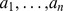 at absolute temperature 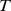, defined by
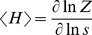
(1)where 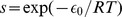 – see [27]. Subsequently, it is possible to plot the *expected helical fraction* 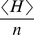 as a function of temperature. Non-cooperative energy models show an approximately linear relation, where the expected helical fraction slowly decreases as temperature increases. In contrast, the plot of expected helical fraction versus temperature for *cooperative* energy models displays a *sigmoidal* shape, where there is an abrupt helix-coil transition from high to low values for the helical fraction that occurs at a *critical temperature* 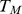.

Polymer theory provides several mathematical models to explain the temperature-dependent *helix-coil transition* for proteins. The simplest polymer model for the helix-coil transition of an 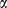-helix is the *non-cooperative* model, where the probability that the each residue is H is independent of the conformation of every other residue. The cooperative, nearest-neighbor model for helix-coil transition, introduced by Zimm and Bragg [28], includes nucleation free energy 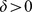 that is applied for each 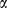-helical segment of contiguous H residues. Finally, the Ising model was introduced by E. Ising in 1925 [29] to explain ferromagnetism, but has subsequently been used to model protein temperature-dependent helix-coil transitions – see, for instance [30]. Progressing from the independent model to the Zimm-Bragg model to the Ising model, each model is increasingly cooperative, thus providing a better fit to the experimental data. See Dill and Bromberg [27] for a more detailed discussion.

**Figure 1:**
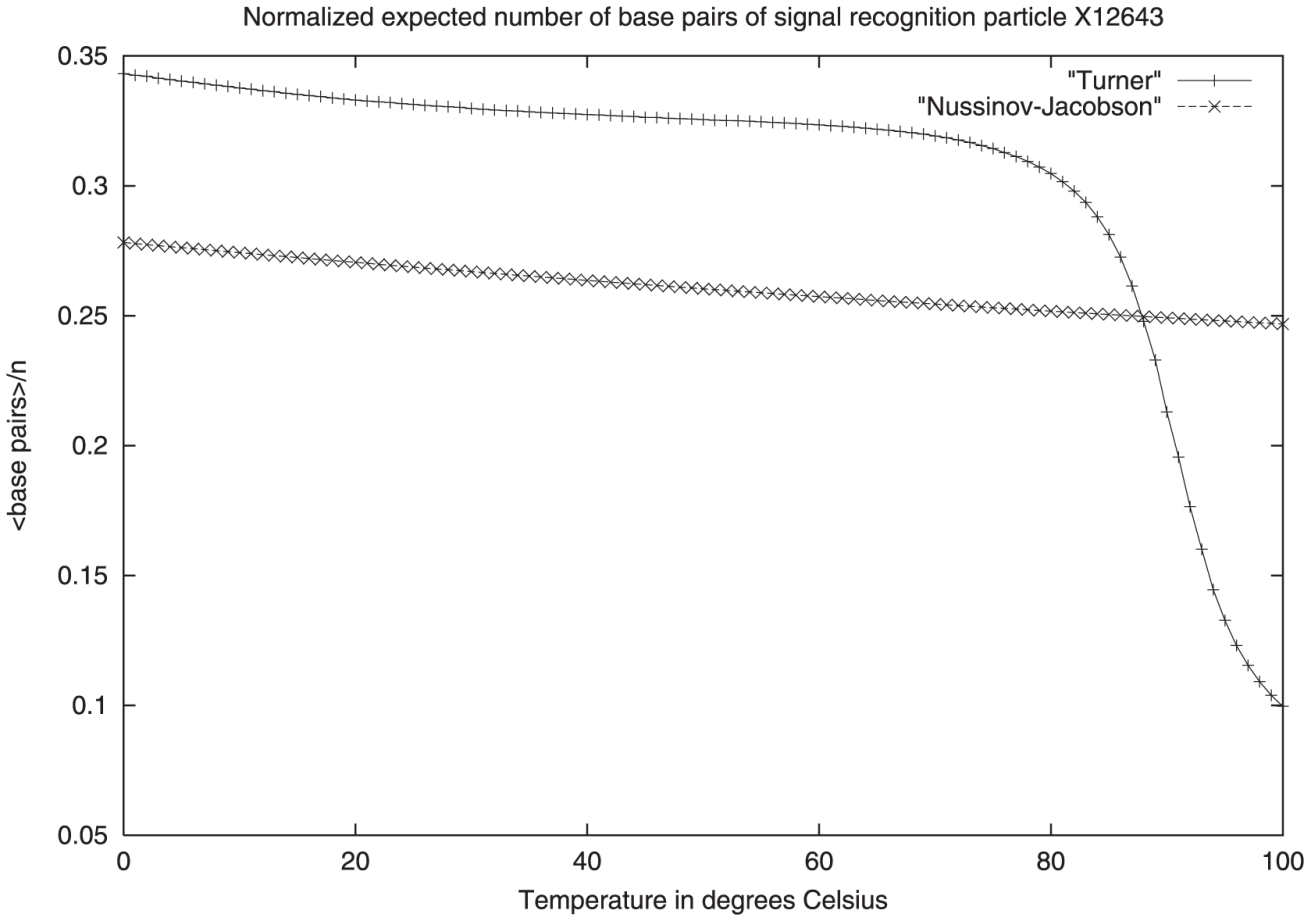
Graph of the expected number of base pairs as a function of temperature for signal recognition particle with Rfam [56] accession number X12643. Temperature in degrees Celsius is given on the 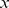-axis, while the expected number of base pairs 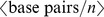, normalized by sequence length 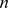, is given on the 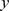-axis. Note the linear dependence on temperature for the non-cooperative Nussinov energy model, in contrast to the sigmoidal dependence on temperature for the cooperative Turner energy model. Data and figure taken from our paper [57].

In the Nussinov energy model [31] for RNA secondary structure, the free energy of a secondary structure 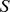 is defined to be 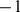 times the number 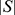 of base pairs of 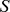; i.e. in the Nussinov model, each base pair contributes an energy of 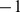, and there is no energy term for entropic considerations. The Turner energy model [13], [32] for RNA secondary structure contains negative free energies for base stacks, which depend on the nucleotides involved, such as the base stacking free energy of 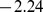 kcal/mol at 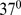
C for 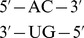 and of 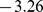 kcal/mol for 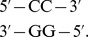. Additionally the Turner energy model contains free energies for various loops (hairpin, bulge, internal loop, multiloop) that include entropic considerations. Clearly, the Turner energy model for RNA secondary structure is analogous to the cooperative, nearest-neighbor model for helix-coil transitions introduced by Zimm and Bragg. Figure 1 contrasts the temperature-dependent cooperativity of the Turner energy model with the temperature-independent non-cooperativity of the Nussinov energy model. The motivation for this paper is to solve the equation: 
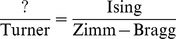
. Though we do not determine the analogue of the Ising model for RNA secondary structure formation, we do introduce an *extended nearest-neighbor* model, also called *triplet* or *next-nearest-neighbor* model, which displays somewhat more cooperativity, as displayed in the sharpness of the transition from folded to unfolded state in a figure shown later in the paper.

### Subsection 1.2: Triplet model

As previously mentioned, the nearest-neighbor energy model [13], [32] assigns free energies for base stacks of the form 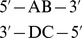 for the formation of a stacked base pair between 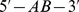 with 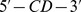. In contrast, the extended-nearest-neighbor triplet model assigns free energies for triplexes of the form 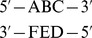 where a stacked triple (two contiguous base stacks) between 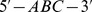 and 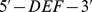. In this case, we expect that the triplet free energy of 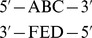 can be approximated by the average of the base stacking free energies for 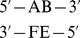 and 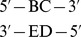; however, we expect the triplet energies to more accurately model the formation of secondary RNA structure.

The extended-nearest-neighbor triplet model for hybridized DNA duplexes and DNA-RNA hybrids was considered in experimental work of D.M. Gray, who in Table 1 of [12] determined the theoretical number of independent hybridized sequences that must be considered in UV absorbance experiments, in order to obtain triplet stacking free energies by least-squares fitting of data. In [33] Gray et al. experimentally determined *in vivo* inhibition parameters for next-nearest-neighbor triplets in the case of antisense DNA – RNA hybridization to inhibit protein expression. In [34], Najafabadi et al. applied a neural network to predict the thermodynamic parameters for the next-nearest-neighbor triplet model, using existent UV absorbance data from the thermodynamic database for nucleic acids, NTDB version 2.0 [35].

**Table 1:** Values of sensitivity and positive predictive value (ppv) for RNAfold and RNAenn with respect to various RNA families.

	RNAfold -d 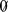		RNAenn (Turner99)		RNAenn (Turner04)	
RNA family	sens	ppv	sens	ppv	sens	ppv
16s	0.3940	0.3326	0.3779	0.3153	0.3099	0.2674
23sd	0.5974	0.5311	0.5527	0.4813	0.4409	0.4003
23s	0.4685	0.3972	0.4453	0.3738	0.3516	0.3061
5s	0.7575	0.6606	0.7319	0.6366	0.5713	0.5093
ec	0.5869	0.5314	0.6184	0.5519	0.5338	0.4982
grp1	0.6625	0.5832	0.5047	0.4650	0.4837	0.4589
grplii	0.6616	0.6409	0.6084	0.5976	0.4555	0.4334
rnap1	0.4051	0.3813	0.3514	0.3221	0.3154	0.3000
rnap2	0.4241	0.4046	0.4935	0.4648	0.3285	0.3187
short	0.4048	0.3400	0.3690	0.3298	0.3155	0.2760
srp	0.7228	0.5632	0.6286	0.4897	0.5677	0.4544
telomerase	0.4285	0.3074	0.3417	0.2404	0.3605	0.2662
tmRNA	0.2248	0.1958	0.1911	0.1622	0.1526	0.1326
trna2	0.4960	0.4697	0.5344	0.5005	0.3962	0.3828
avg	0.5213	0.4575	0.4866	0.4279	0.4020	0.3678

Sensitivity is the ratio of number of correctly predicted base pairs divided by the number of base pairs in the native structure; positive predictive value is the ratio of the number of correctly predicted base pairs divided by the number of base pairs in the predicted structure. Since RNAenn currently does not include energy contributions for dangles (single stranded, stacked nucleotides), RNAfold was used without dangles (version 1.8.5 with -d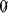 flag). To our knowledge, there has not been a careful benchmarking of structure prediction accuracy between the Turner 1999 energy model and the newer Turner 2004 energy model, though it is interesting to note that RNAenn has better structure prediction when using Turner 1999 for base stacking. Overall, it is clear that RNAfold outperforms RNAenn (Turner99), although a few cases, such as **ec** and **rnap2** RNAenn have better sensitivity. Nevertheless, we expect much better performance in the future when our triplet and base stacking energy terms have been refined by using knowledge-base potentials. The database of RNA structures in this benchmarking set comes from a data collection of D.H. Mathews (personal communication), which derives from published databases [26], [54], etc. See [55] for a citation of original data sources.

Though at present there are no experimentally determined free energies for triplet stacking, Binder et al. [36] did show a strong correlation between microarray fluorescence intensities and DNA-RNA base stacking free energies of Sugimoto et al. [37]. More precisely, Binder et al. showed that linear combinations of triple-averaged probe sensitivities provide nearest-neighbor *sensitivity* terms, that rank in similar order as the base stacking free energy parameters for DNA-RNA in solution [37]. It is our hope that future improvements in RNAseq, microarray or other technologies will ultimately furnish experimentally determined triplet and even 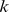-tuple stacking free energies. New triplet free energies could immediately be incorporated into our algorithms, and it is tedious, but clear how one can modify our algorithms to handle 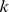-tuple free energies.

### Subsection 1.3: Plan of the paper

In this paper, we describe the first algorithms to compute the partition function and minimum free energy structure for single-stranded RNA, with respect to the full *next-nearest-neighbor triplet* energy model for RNA. In the *Introduction*, we gave the motivation for this work, coming from the Zimm-Bragg and Ising models in biopolymer theory. The plan for the remainder of the paper is as follows. In the *Results* section, Section 2.1 gives the notation and definitions needed for the sequel, while Section 2.2 presents the extended nearest neighbor model and method used to obtain energy parameters. In the *Discussion*, we give secondary structure benchmarking results for the nearest neighbor (NN) and extended nearest neighbor (ENN) energy models. Additionally, the cooperativity of folding is compared with both energy models. In the *Methods* section, Section 3.1 [resp. Section 3.2] presents recursions for the partition function [resp. minimum free energy structure] computation. In addition to the software RNAnn and RNAenn developed for this paper, we use Vienna RNA Package RNAfold [18], RNAstructure [38], and mfold [9]. As illustration for the cooperativity of folding, we compare melting curves for two small nucleolar RNAs (snoRNA), with respect to the NN and ENN energy models; additional melting curves are available on the web server http://bioinformatics.bc.edu/clotelab/RNAenn/. These results suggest that the the extended nearest-neighbor energy model may lead to more cooperative folding than does the nearest-neighbor model, which was our motivation to study the ENN energy model.

The goal of this paper is to describe the non-trivial RNAenn algorithms, which are implemented in C/C++. Our work points toward a future potential improvement in RNA secondary structure prediction, either by incorporating triplet knowledge-based potentials or experimentally inferred extended nearest-neighbor free energy parameters.

## Results: Extended nearest neighbor model algorithms

Assume that 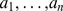 is a given RNA sequence. In this section, we describe pseudocode for the partition function and minimum free energy computation for an extended nearest neighbor model. Although our software, RNAenn, does depend on the exact values of the extended nearest-neighbor energy parameters, the description of the algorithms does not.

### Subsection 2.1: Notation and definitions

Let 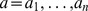 be an arbitrary RNA sequence, and let 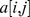 denote the subsequence 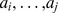. A *secondary structure* 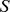 for a given RNA sequence 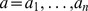 is a set of *base pairs* 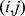, 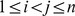, such that (1) 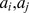 forms a Watson Crick AU, UA, GC, CG or wobble GU, UG pair; (2) each base is paired to at most one other base, i.e. 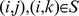 implies that 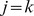, and 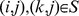 implies that 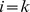; (3) there are no pseudoknots in 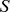, where a pseudoknot consists of base pairs 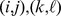 where 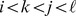; (4) each hairpin loop has at least 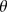 unpaired bases; i.e. 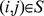 implies that 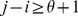.

In software such as mfold [9], Unafold [39], RNAfold [40], and RNAstructure [38], the parameter 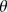, denoting the minimum number of unpaired bases in a hairpin loop, is taken to be equal to 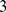, due to steric constraints of RNA molecules.

**Figure 2:**
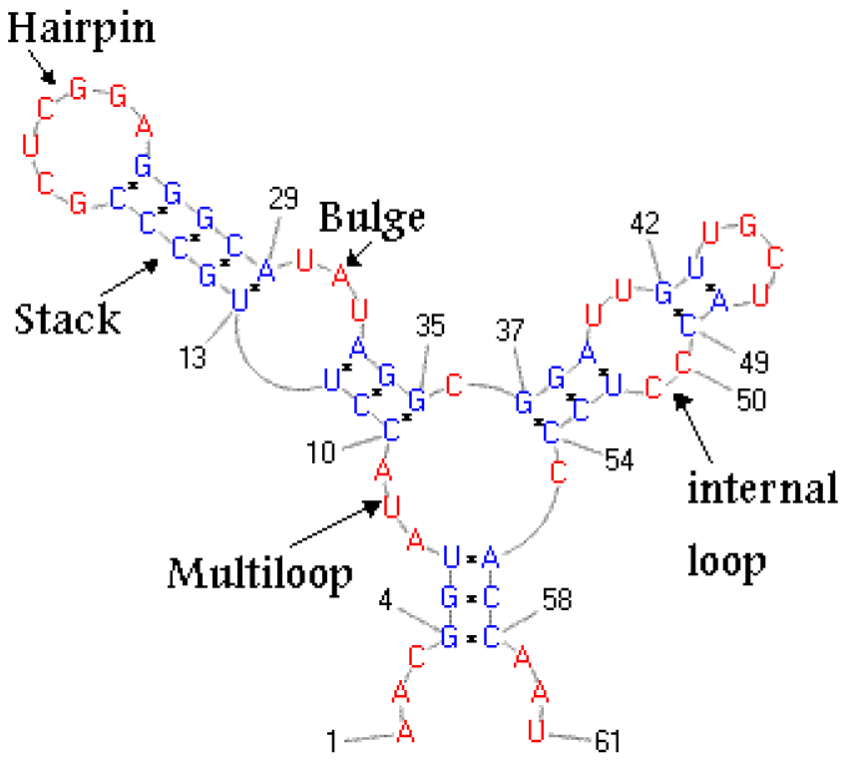
Depiction of elements of RNA secondary structure for which experimentally determined free energy parameters are available. In this 61 nt RNA, the hairpin loop closed by base pair between nucleotides at position 43 and 48 is known as a *tetraloop*, or hairpin loop of size 4. Similarly, the hairpin loop of size 7 is closed by a base pair between nucleotides at positions 17 and 25. Free energy parameters for bulges and internal loops (two-sided bulges, not shown in the figure) are available, while an affine approximation is used for the free energy of a multiloop or junction.

The nearest-neighbor and extended nearest-neighbor triplet models are additive energy models that entail free energy values for *loops*, as explained in [41]. A *hairpin* in a secondary structure 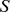 is defined by the base pair 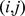, where 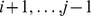 are unpaired. A *left bulge* in 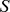 is defined by the two base pairs 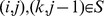, where 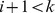 and 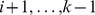 are unpaired. A *right bulge* in 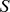 is defined by the two base pairs 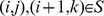, where 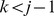 and 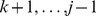 are unpaired. An *internal loop* in 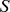 is defined by the two base pairs 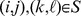, where 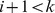 and 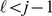 and 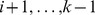 and 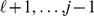 are unpaired. Finally, a 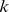-way junction, or multiloop with 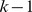 components, is defined by the closing base pair 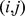 and 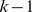 inner base pairs 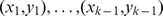, where 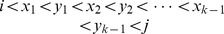, and the nucleotides in intervals 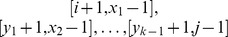 are all unpaired. See Figure 2 for an illustration.

Given the RNA nucleotide sequence 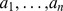, we use the notation 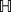 to denote the free energy of a hairpin, 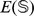 to denote the free energy of a stacked base pair, 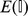 to denote the free energy of an internal loop, 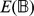 to denote the free energy of a bulge, while the free energy 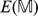 for a multiloop containing 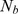 base pairs and 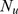 unpaired bases is given by the affine approximation 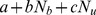. The free energy 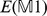 of a multiloop having exactly one component is then given by 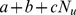.

For RNA sequence 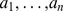, for all 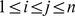, the partition function 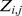 is defined by 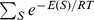, where the sum is taken over all secondary structures 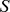 of 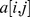, 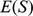 is the free energy of secondary structure 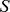, 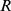 is the universal gas constant with value 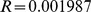 kcal/mol^−1^ K^−1^, and 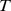 is absolute temperature. In the Zuker [9], [18], [38] and McCaskill [19] algorithms, 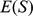 is the Turner nearest neighbor energy model; in contrast, when discussing the extended nearest-neighbor energy model, we use 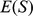 to denote the triplet energy model.

Given an RNA sequence 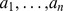, in order to compute the partition function 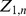 [resp. minimum free energy 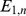] for 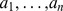, we need inductively to determine the partition function 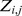 [resp. minimum free energy 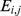] for all smaller subsequences 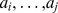. In so doing, we need to know which structures involve a triple stack 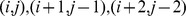, which structures involve only a stacked pair 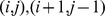, and which structures involve a base pair 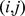 which closes a loop region. This is accomplished by terms 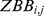 [resp. 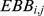]. Moreover, the Turner energy model stipulates that a base pair 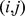, which closes a left bulge of size 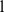, as in 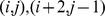, or a right bulge of size 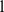, as in 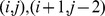, is considered to stack on the subsequent base pair. This consideration requires the introduction of special terms 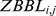, 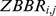 [resp. 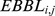, 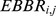]. With that, we have the following definition.

### Definition 1 (Energies and partition function for triplet loop model)

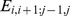
 *denotes the base stacking free energy from the NN model, while* 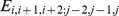 *denotes the triplet stacking free energy from the ENN model.*

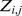
 *: partition function over all secondary structures of* 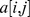.

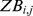
: *partition function over all secondary structures of* 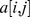, *which contain the base pair* 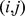.

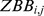
: *partition function over all secondary structures of* 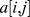 *, which contain the base pairs* 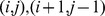.

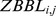
: *partition function over all secondary structures of* 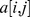, *which contain the base pairs* 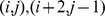.

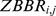
: *partition function over all secondary structures of* 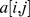, *which contain the base pairs* 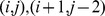.

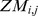
: *partition function over all secondary structures of* 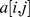, *subject to the constraint that* 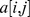 *is part of a multiloop and has at least one component.*

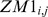
: *partition function over all secondary structures of* 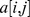, *subject to the constraint that* 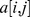 *is part of a multiloop and has at exactly one component. Moreover, it is required that* 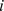 *base-pair in the interval* 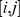; *i.e.* 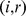 *is a base pair, for some* 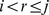.

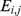
: *minimum free energy over all secondary structures of* 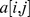.

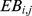
: *minimum free energy over all secondary structures of* 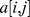, *which contain the base pair* 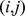.

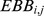
: *minimum free energy over all secondary structures of* 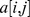, *which contain the base pairs* 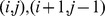.

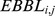
: *minimum free energy over all secondary structures of* 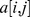, *which contain the base pairs* 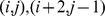.

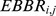
: *minimum free energy over all secondary structures of* 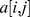, *which contain the base pairs* 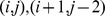.

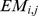
: *minimum free energy over all secondary structures of* 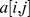, *subject to the constraint that* 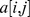 *is part of a multiloop and has at least one component.*

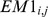
: *minimum free energy over all secondary structures of* 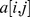, *subject to the constraint that* 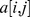 *is part of a multiloop and has at exactly one component. Moreover, it is required that 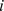 base-pair in the interval* 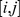; i.e. 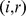 *is a base pair, for some* 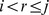.

Details for the recursions necessary to compute the ENN minimum free energy secondary structure and ENN partition function are given in the *Methods* section.

### Subsection 2.2: Extended nearest-neighbor energy model ENN-13

Here we describe details for the extended nearest-neighbor energy model parameters, which we denote by ENN-13, since our code RNAenn was completed in 2013.

Though some related experimental work has been done, especially by D.M. Gray and co-workers [11], [12], [33], there are no available experimentally determined parameters for triplet stacking. Rather than using the triplet stacking free energy parameters INN-48 [34], which are incomplete since GU-wobble pairs were not included, we instead infer RNA triplet stacking free energies by a novel use of Brown's algorithm [42], which computes the *maximum entropy* joint probability distribution that is consistent with given user-specified marginal probabilities. Though Brown's algorithm has been used by C. Burge to predict intron-exon splice sites in the human gene finder, *GenScan* [43], this appears to be the first use of Brown's algorithm to infer free energy parameters.

#### Brown's algorithm for maximum entropy joint distribution

In [42], D.T. Brown described an efficient algorithm to compute the *maximum entropy* joint probability distribution given certain marginal probabilities, where we recall that the entropy of a joint probability distribution 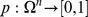 is defined by 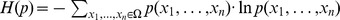. Although the algorithm was correct, there was an error in Brown's proof of correctness, which was subsequently repaired by Ireland and Kullback [44], who additionally showed that the maximum entropy distribution is *not* the maximum likelihood distribution.

Suppose that 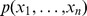 is a given joint probability distribution on 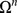, where 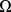 is the alphabet 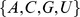 of RNA nucleotides. Recall that a marginal probability distribution 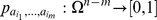 is defined by the projection
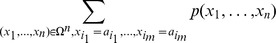



Given an integer 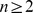, a value 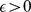, and a set of arbitrary marginal probabilities 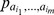 the idea is to initialize 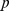 to the uniform distribution, then repeatedly update 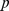 so that it has the correct currently considered marginal.

**Algorithm 1 (Brown's algorithm** [**42**] **)** Input: *Finite sample space* 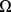 *, integer* 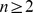 *,* 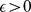 *, set of arbitrary (target) marginal probabilities* 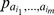. Ouput: *Maximum entropy joint probability distribution* 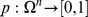 *having given marginals (i.e. within* 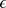 *of target marginals).*

Idea:
   initialize 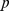 to the uniform distribution
   repeat
      for each target marginal 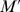

         compute current marginal 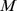 of 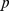

**Figure 3:**
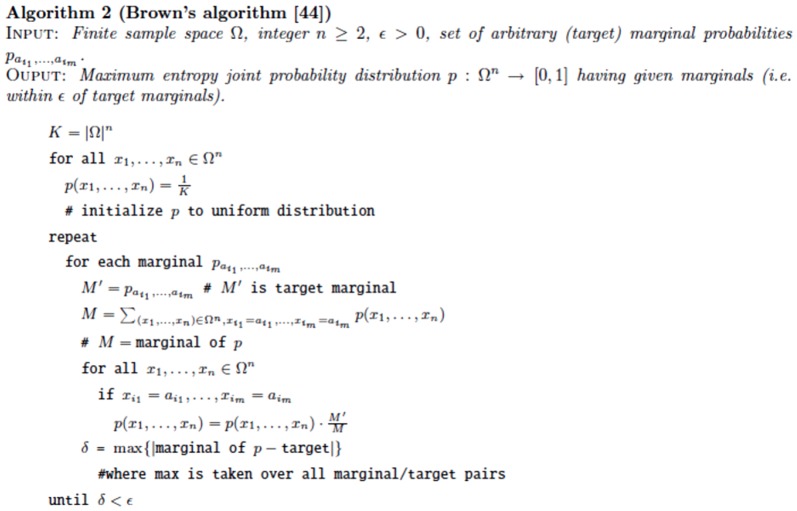
Pseudocode for Brown's algorithm.

         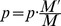

   until 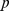 has all the desired marginals
See Figure 3 for more detailed pseudocode of Brown's algorithm.

#### Conversion between free energies and probabilities

To compute triplet stacking free energies, base stacking free energies from Turner 1999 [or alternatively Turner 2004] energy model are converted to marginal probabilities in the following manner. Given a triplet stack
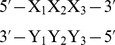
where the outermost base pair occurs at 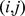, let 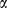 denote the outermost base pair 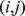 with nucleotides 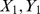, let 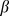 denote the middle base pair 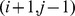 with nucleotides 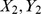, and let 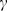 denote the innermost base pair 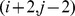 with nucleotides 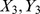. It is a well-known principle, first proved by Jaynes [45] and subsequently exploited in protein threading algorithms [46], [47], that a representative database of biomolecular sequences and structures has the property that motif occurrences are Boltzmann distributed – i.e. motif frequencies are of the form 
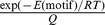
, where the partition function 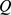 is the sum of Boltzmann factors of all motifs. For this reason, we define the *left*, *middle* and *right marginal* probabilities of stacked base pairs by:
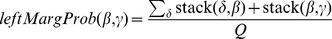


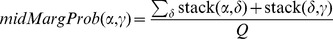


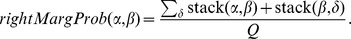
where 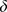 ranges over the six base pairs GC, CG, AU, UA, GU, UG, stack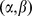 denotes base stacking free energies from the Turner 1999 model [or alternatively Turner 2004 model], and the partition function
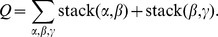



In words, the left/middle/right marginal probability is defined by the quotient of the sum over all six base pairs in the left/middle/right position, while fixing the remaining two base pairs, divided by the partition function. We then apply Brown's algorithm to compute the joint probability distribution 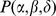 for all base pairs 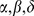, and thus obtain triplet stacking free energies
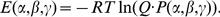
(2)


An additional potential advantage of the extended nearest neighbor energy model is that the MFE structure is perhaps less likely to have isolated base pairs, than when using base stacking free energies. In particular, Bompfunewerer et al. [48] described an 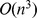 algorithm to compute the MFE structure and partition function over all *canonical* secondary structures; i.e. those having no *isolated base pairs*, where an isolated base pair 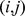 has no adjacent base pair 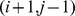 or 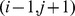. Bompfunewerer et al. stated that preliminary studies indicated that canonical MFE structure prediction is both faster and more accurate. In [49] we provided theoretical reasons for the computational speed-up, by using complex analysis to prove that the asymptotic number of canonical secondary structures is 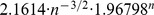, compared to the much larger number 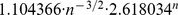 of all secondary structures, a result obtained in [50] by a different method.

Apart from the triplet stacking energy, ENN-13 contains free energies for base stacks (used only at stem ends), hairpins, bulges, internal loops and multiloops from the Turner NN model – here, the user may choose between the Turner 1999 parameters and the Turner 2004 parameters, the former taken from Vienna RNA Package 1.8.5 and the latter taken from the Nearest Neighbor Data Base (NNDB) http://rna.urmc.rochester.edu/NNDB/ [15]. For readers interested in the exact nature of the NN energy parameters, we recommend the excellent overview by Zuker et al. [58].

## Discussion

**Figure 4:**
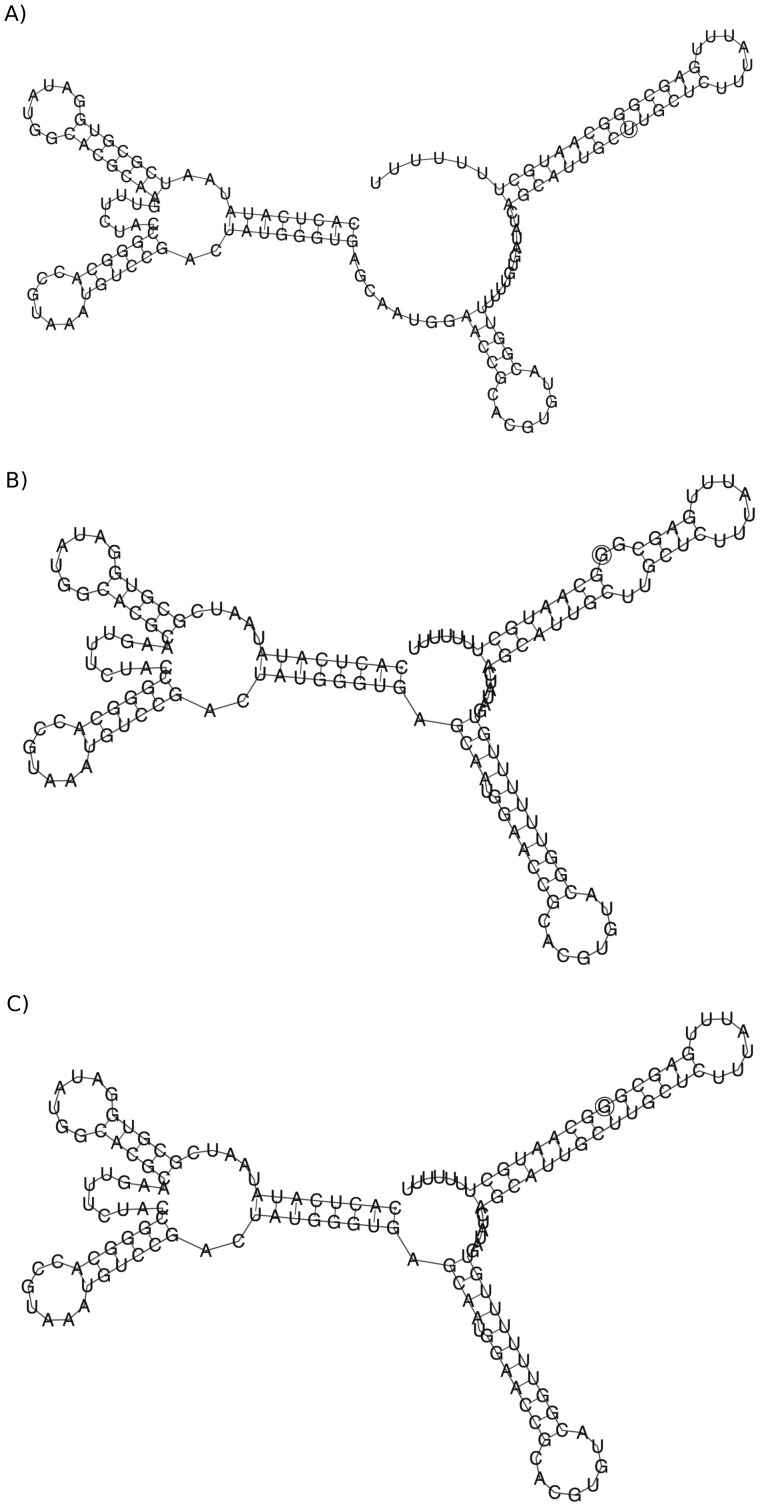
Secondary structure of the XPT guanine riboswitch from *Bacillus subtilis*, with experimentally determined 148 nt sequence CACUCAUAUA AUCGCGUGGA UAUGGCACGC AAGUUUCUAC CGGGCACCGU AAAUGUCCGA CUAUGGGUGA GCAAUGGAAC CGCACGUGUA CGGUUUUUUG UGAUAUCAGC AUUGCUUGCU CUUUAUUUGA GCGGGCAAUG CUUUUUUU taken from Wakeman et al. [58] **.** *(Left)* Gene off structure, determined by in-line probing – see [59] for X-ray structure of aptamer, which is consistent with the secondary structure. *(Center)* Minimum free energy (MFE) structure, determined by RNAnn, our implementation of the nearest-neighbor energy model. This structure is identical to the MFE structures computed by Vienna RNA Package RNAfold [18], RNAstructure [38], and mfold [9]. *(Right)* Minimum free energy (MFE) structure, determined by RNAenn, our implementation of the extended nearest-neighbor energy model. The only difference with the nearest-neighbor MFE structure lies in two missing GU base pairs 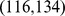, 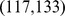 indicated by a circle.

There are some deviations between the MFE structure computed for the ENN model, compared with the nearest-neighbor (NN) model. In particular, Figure 4 shows the secondary structure for the XPT riboswitch from *Bacillus subtilis*, obtained by experimental in-line probing (left panel), minimum free energy structure computation for the NN model (middle panel) and minimum free energy structure computation for the ENN model (right panel). The MFE structure for the NN model was identical, using four different software packages: mfold [9], RNAfold [40], RNAstructure [38] and our own program RNAnn for the nearest-neighbor model. Our software RNAenn for the ENN model differs from the NN minimum free energy structure, only by missing two GU-wobble base pairs at positions 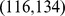, 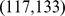. Adjacent wobble pairs are energetically weak, so we do not view this as a failure of our software, but rather the need for additional scrutiny of the ENN energy parameters. Specifically, in the future, we intend to include a dependence on the heat capacity 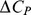 as proposed by M. Bon [17], and knowledge-based potentials [16], [17]. By such energy re-parametrization, we expect to improve the sensitivity values reported in Table 1.

**Figure 5:**
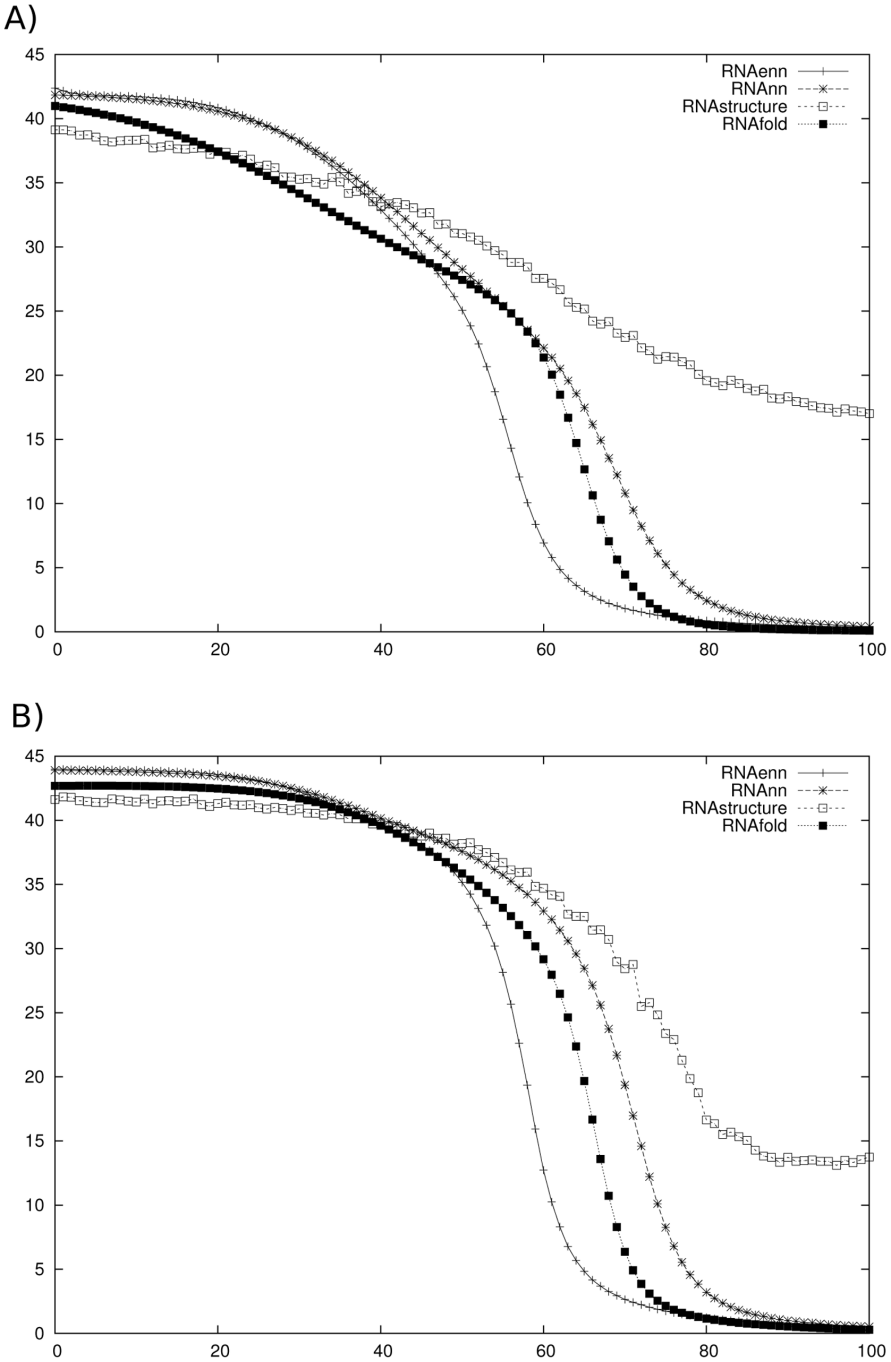
Melting curves for two small nucleolar RNAs (snoRNA) from family RF00158 from Rfam version 9.0 [56]. For each RNA sequence, over a range of temperatures, temperature-dependent base pair probabilities were computed using four different software packages: RNAenn, RNAnn, version 1.8.5 of RNAfold [40] and RNAstructure [38]. The software RNAenn (RNA extended nearest-neighbor) is our implementation of the algorithms described in this paper, while the software RNAnn (RNA nearest-neighbor) is our implementation of the following algorithms: Zuker's minimum free energy structure algorithm [51], McCaskill's partition function algorithm [19], and the Ding-Lawrence sampling algorithm [22]. Each algorithm was run without dangle or coaxial free energies. At each temperature 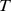, for each algorithm, the expected number 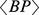 of base pairs was computed as 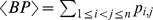; for each algorithm, the collection of such 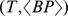 points generates a melting profile obtained by that algorithm. *(Left)* Melting curves for the 72 nt small nucleolar RNA (snoRNA) from *Ornithorhynchus anatinus* (platypus) with GenBank accession code AAPN01359272.1/4977–5048 and sequence given by AGCACAAAUG AUGAGCCUAA AGGGACUUAA UACUGAAACC UGAUGUAACU AAAUAAUAUA UGCUGAUCGU GC *(Right)* Melting curves for the 69 nt small nucleolar RNA (snoRNA) from *Otolemur garnetti* (small-eared galago) with GenBank accession code AQR01179445.1/1047–1115 and sequence given by GGCACAAAUG AUGAAUGACA AGGGACUUAA UACUGAAACC UGAUGUUACA UUACAAUGUG CUGAUGUGC.

Our next-nearest-neighbor *triplet* energy model appears to lead to somewhat more *cooperative* folding than does the nearest neighbor energy model, as indicated by sharper sigmoidal transition in the melting curves obtained by RNAenn, compared to melting curves obtained by RNAfold and RNAstructure – see Figure 5. Here, melting curves were computed in the following manner. For each RNA sequence, over a range of temperatures, temperature-dependent base pair probabilities were computed. At each temperature 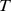, for each algorithm, the expected number 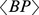 of base pairs was computed by 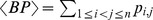. For each algorithm, the collection of all points with 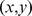 coordinates given by 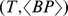 generates a melting profile.

## Methods

The top level recursion in the computation of the partition function [resp. minimum free energy structure] is identical to that of McCaskill's algorithm [19] [resp. Zuker's algorithm [51]]. The technical difficulty lies in a kind of “2-look-ahead” strategy, to determine if a base pair 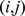 not only stacks onto the adjacent base pair 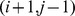, but the latter also stacks onto the base pair 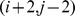. This leads to technical issues, including a special treatment for bulges of size 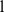, since these are considered to stack on the following base pair.

### Subsection 3.1: Partition function algorithm

**Figure 6:**
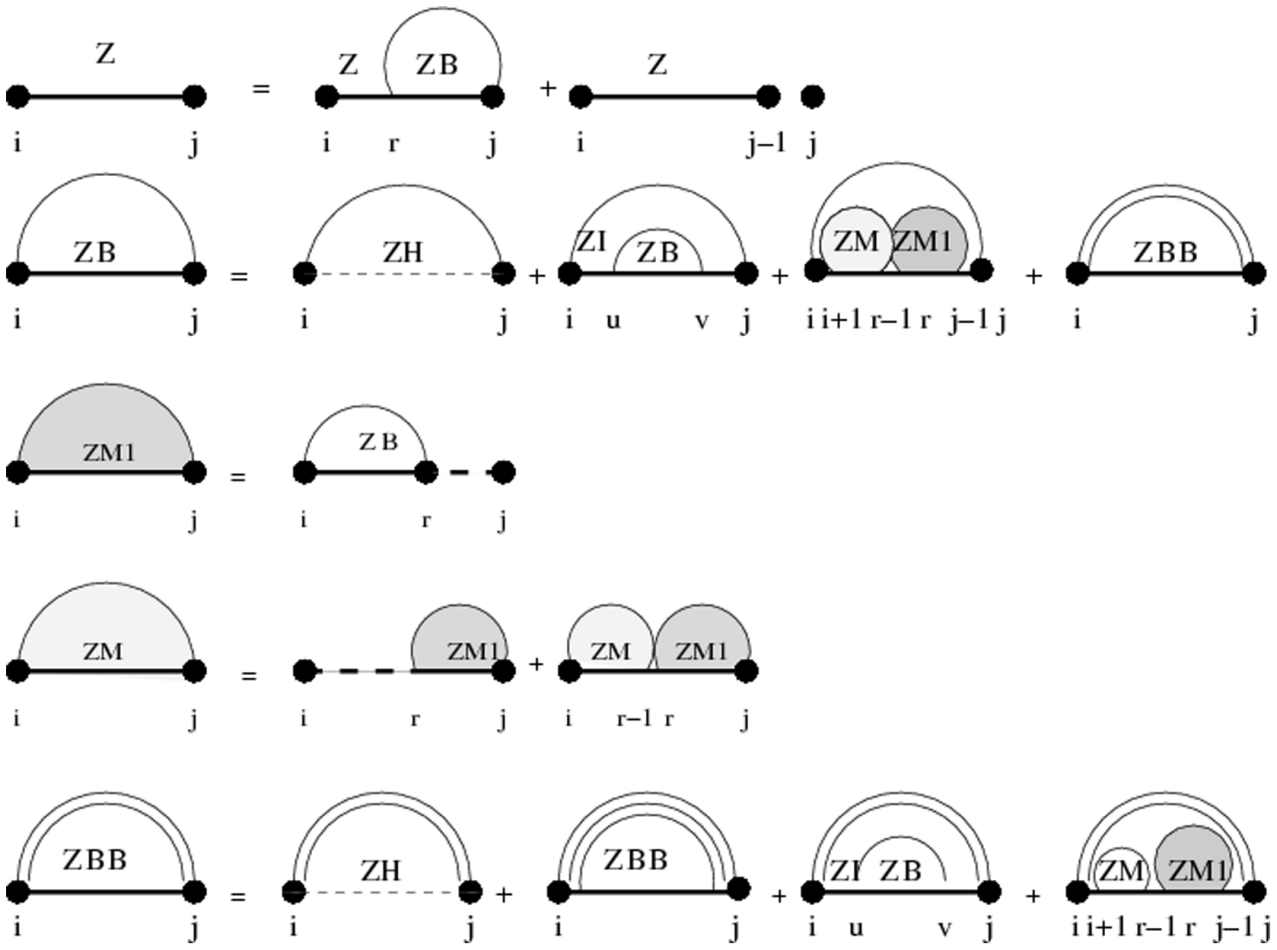
Feynman diagram to pictorially describe recursions described in this proposal for partition function with respect to extended nearest neighbor model. For simplicity, this diagram depicts 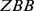, but not 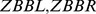, which correspond to a special treatment for particular left/right bulges of size 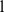, that are treated as stacked base pairs.

This section presents the recursions to compute the partition function in the extended nearest-neighbor energy model. Figure 6 depicts the recursions as a Feynman diagram. (For simplicity, the Feynman diagram in Figure 6 depicts 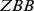, but not 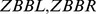, which correspond to a special treatment for particular left/right bulges of size 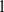, that are treated similarly to stacked base pairs.) The unconstrained partition function 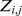 for 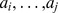 is defined below. Note that the recursions for 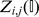 entail a maximum internal loop of size 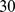, which follows the Vienna convention to reduce run time of the algorithm to 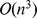; however, our implementation actually uses the more complicated treatment of Lyngsø et al. [52], which ensures a cubic run time while not arbitrarily bounding the maximum size of internal loops. A similar remark applies to the treatment of internal loops and bulges of size 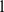 in Section 3.2.
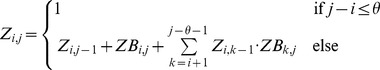



We now in turn describe the partition functions 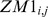 for a multiloop having a single component, 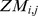 for a multiloop having one or more components, 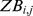 where 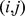 pair together, and for 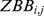 where 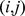 and 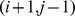 pair together.
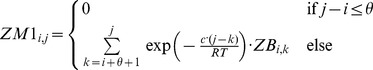


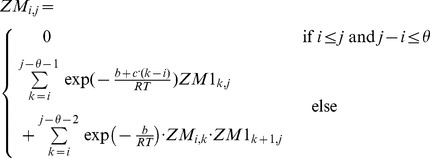


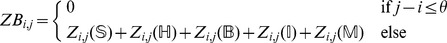
where
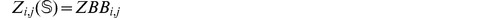


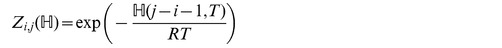


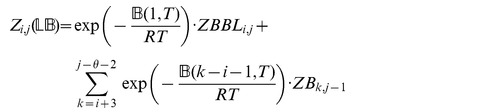


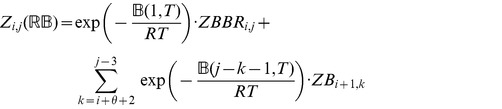


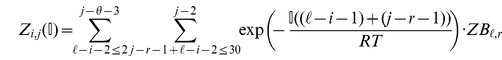


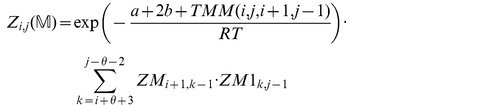


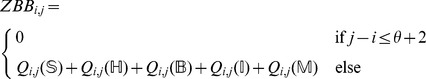
where
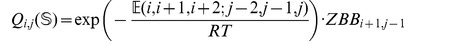


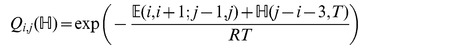


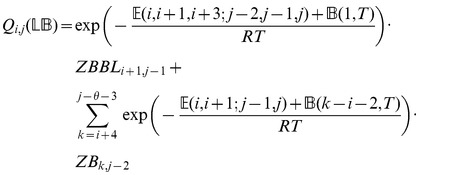


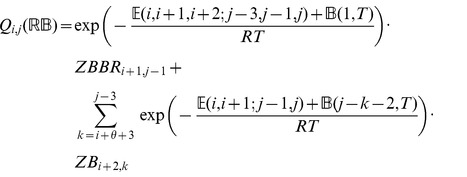


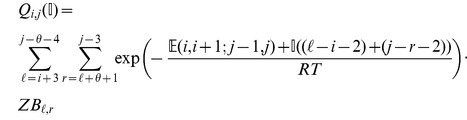


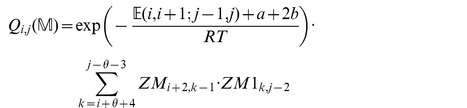


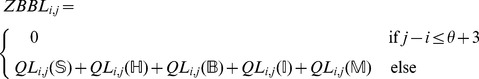
where
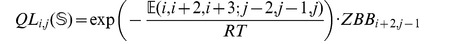


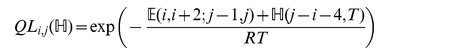


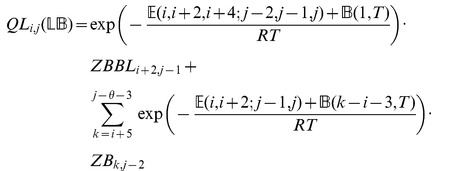


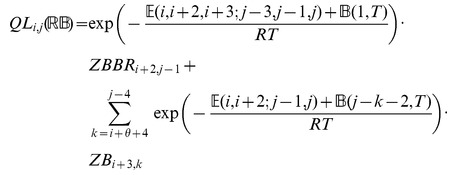


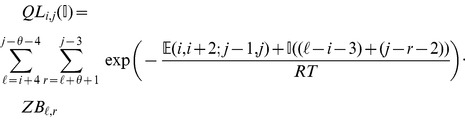


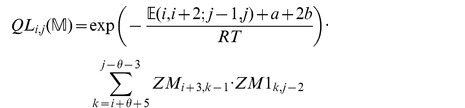


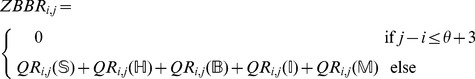
where
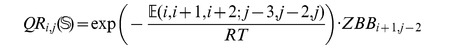


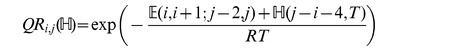


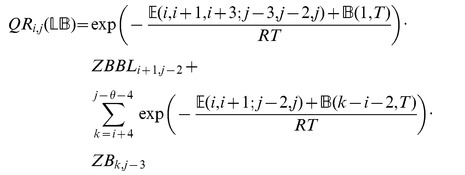


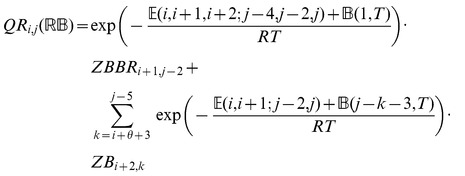


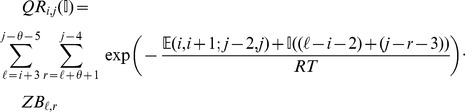


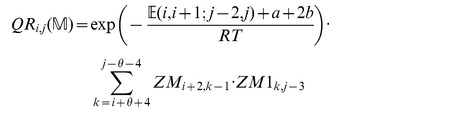



### Subsection 3.2: Minimum free energy algorithm

Assume that 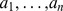 is a given RNA sequence. Throughout this section, we let 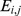 denote the minimum free energy of 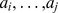, which is computed and stored in arrays by a dynamic programming algorithm corresponding to the following recursions. Once 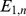 is computed, then the minimum free energy structure can be computed by tracebacks. The following recursions are obtained from those in the previous section, by systematically replacing sum by minimum, product by sum and Boltzmann factor by energy.
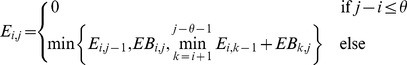


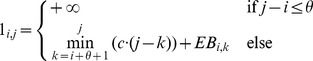


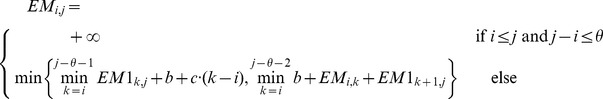


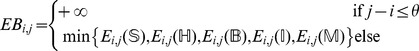
where
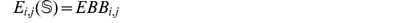


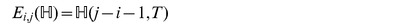


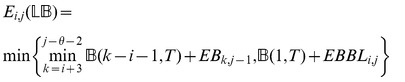


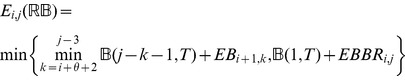


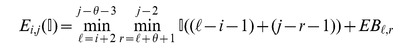


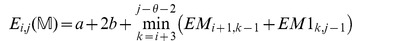


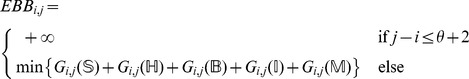
where
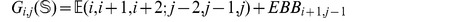


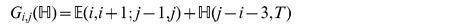


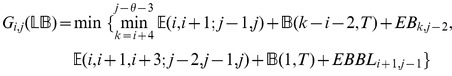


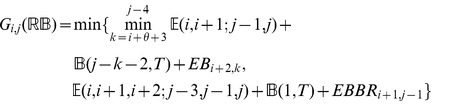


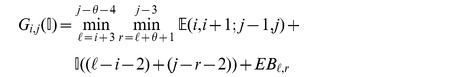


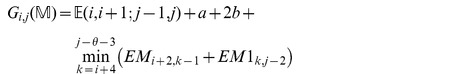


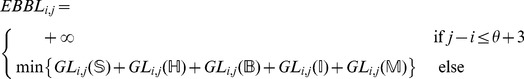
where
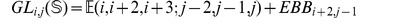


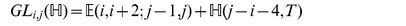


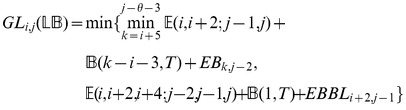


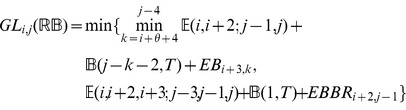


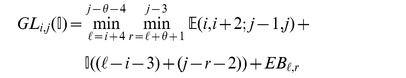


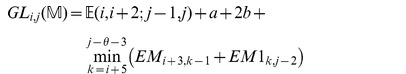
dlinsteadt
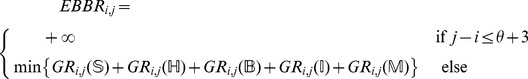
where
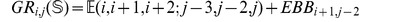


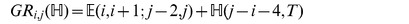


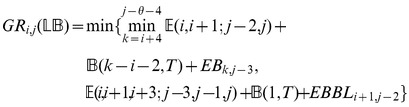


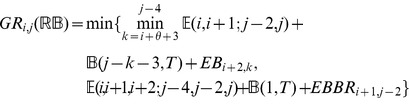


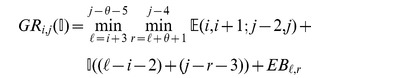


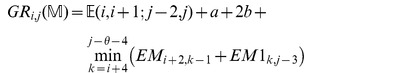



## Conclusion

In this paper, we have introduced a new energy model ENN for RNA secondary structure prediction and implemented it in a tool called RNAenn along with new energy parameters for triplet stacking inferred using Brown's algorithm. RNAenn is implemented in C/C++, without any function calls or dependence on other programs, such as mfold [9], RNAfold [18], and RNAstructure [38]. Recursions from the partition function have been cross-checked by setting free energy parameters to zero, in which case the program returns the number of secondary structures, which can be determined by independent simpler methods.

It is known from experimental work of Silverman and Cech on Tetrahymena group I intron P4–P6 domain [53] that RNA folds cooperatively. The melting curves in Figure 5 demonstrate that our ENN model leads to somewhat more *cooperative* folding than does the nearest neighbor energy model, in the same manner that the melting curves of Figure 1 demonstrate that the nearest neighbor energy model leads to more cooperative folding than the simple Nussinov energy model. For this reason, we feel that RNAenn supports a mathematical model that better reflects the experimental data concerning cooperativity of RNA folding.

From the benchmarking comparison in Table 1, it is clear that triplet stacking free energy parameters need further refinement to produce better agreement with RNA secondary structures, as determined by comparative sequence alignment or X-ray structure. This situation is not unlike the situation with nearest neighbor software mfold, RNAfold, which over the years underwent a series of refinements, with the introduction of additional energy parameters (energy parameters for particular triloops, tetraloops, bulges of size one, etc.). At the present time, software such as Unafold, RNAfold, RNAstructure remain state-of-the-art for RNA secondary structure prediction. However, in future work, we plan to optimize the triplet stacking energy parameters, by using knowledge-base potential as in the work [16], [17] for the nearest neighbor model.
